# Dietary capsaicin and antibiotics act synergistically to reduce non-alcoholic fatty liver disease induced by high fat diet in mice

**DOI:** 10.18632/oncotarget.16975

**Published:** 2017-04-08

**Authors:** Jingjuan Hu, Haihua Luo, Yong Jiang, Peng Chen

**Affiliations:** ^1^ State Key Laboratory of Organ Failure Research, Guangdong Provincial Key Laboratory of Proteomics, Department of Pathophysiology, Southern Medical University, GuangZhou, China

**Keywords:** antibiotics, capsaicin, high fat diet, non-alcoholic fatty liver disease, Pathology Section

## Abstract

The prevalence of non-alcoholic fatty liver disease is increasing rapidly worldwide. However, effective strategies for combating high-fat diet (HFD) induced obesity, fatty liver and metabolic disorder are still limited, and outcomes remain poor. In the present study, we evaluated the combined actions of dietary capsaicin and antibiotics on HFD-induced physiological abnormalities in mice. C57BL/6 male mice were fed with HFD (60% calories from fat) for 17 weeks, and the resultant pathophysiological effects were examined. Antibiotic treatment markedly attenuated gut inflammation and leakiness induced by HFD, whereas capsaicin showed limited effects on the gut. However, dietary capsaicin significantly increased PPAR-α expression in adipose tissue, while antibiotics had no such effect. Animals treated with a combination of capsaicin and antibiotics had the smallest body weight gain and fat pad index, as well as the lowest hepatic fat accumulation. Combination treatment also maximally improved insulin responsiveness, as indicated by insulin tolerance tests. These results suggest the co-treatment of capsaicin and antibiotics, a novel combination strategy, would play synergistically to attenuate the HFD-induced obesity, fatty liver and metabolic disorder.

## INTRODUCTION

The prevalence of non-alcoholic fatty liver disease (NAFLD) induced by long-term fat loading, is increasing rapidly worldwide and has become a heavy health and economic burden [1–2]. Conventional treatments to curb this disease have not been successful due to its complicated pathogenesis [3]. The progression of HFD-induced NAFLD involves multiple-organ interaction, of which the intestine and white adipose tissue are the two main targets [4–5]. HFD results in intestinal abnormalities such as inflammation, dysbiosis, and leakiness. These effects are believed to combine to further cause fatty liver and adipose tissue damage, and promote obesity and diabetes [6–8]. Depletion of gut microbiota is reported to attenuate HFD induced obesity and diabetes [9–10]. On the other hand, white adipose tissue status also plays an important role in obesity progression [11]. Elevated expression of key genes like PPARs promotes white adipose tissue browning and reduces HFD elicited abnormalities [12–13].

Capsaicin, the natural occurring active component of chili peppers, has a demonstrated anti-obesity effect. Recent reports indicate that capsaicin reduced the body weight gain, hepatic lipid accumulation and insulin resistance induced by HFD [14–15]. The adipose tissue may be the main target of capsaicin. Based on the above observations, we decided to test whether capsaicin and antibiotics co-treatment would exhibit beneficial effects for both intestine and adipose tissue during HFD.

## RESULTS

### HFD altered TRPV1 expression in the perigonadal visceral adipose tissue

**Figure 1:**
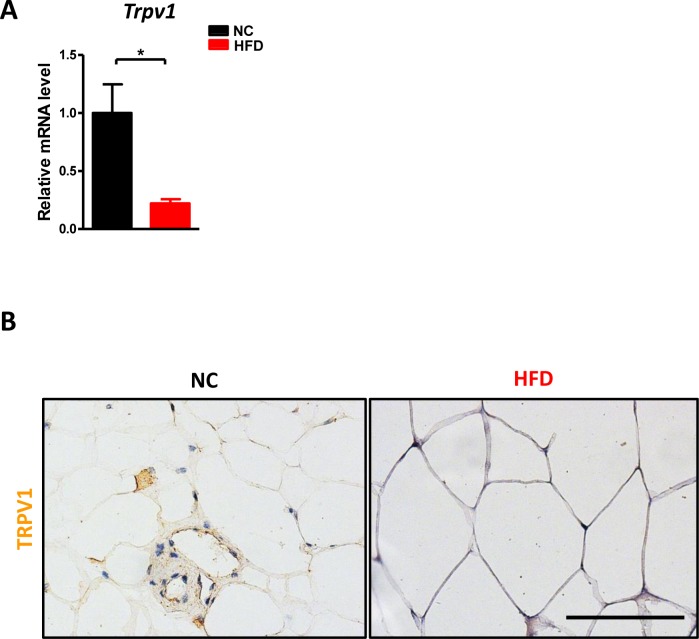
HFD reduced TRPV1 expression in adipose tissue C57BL/6 male mice were fed with a normal chow diet (*n* = 6) or high fat diet (*n* = 10) for 17 weeks. **A**. The mRNA level of *Trpv1* in the perigonadal visceral adipose tissue was detected. **B**. The immunohistochemistry staining for TRPV1. Scale bar, 100 μm. Results are expressed as mean ± SEM. *p < 0.05.

HFD has been reported to reduce TRPV1 expression in the visceral adipose tissue [16]. We examined the expression of TRPV1 which serves as the receptor for capsaicin [17–18] in the adipose tissue to confirm their association. TRPV1 mRNA (Figure 1A) and protein (Figure 1B) levels were dramatically decreased after chronic HFD in perigonadal visceral adipose tissue. Our data confirmed that the capsaicin-associated pathway may influence HFD induced abnormalities.

### HFD leads to enteric dysbiosis in mice

**Figure 2:**
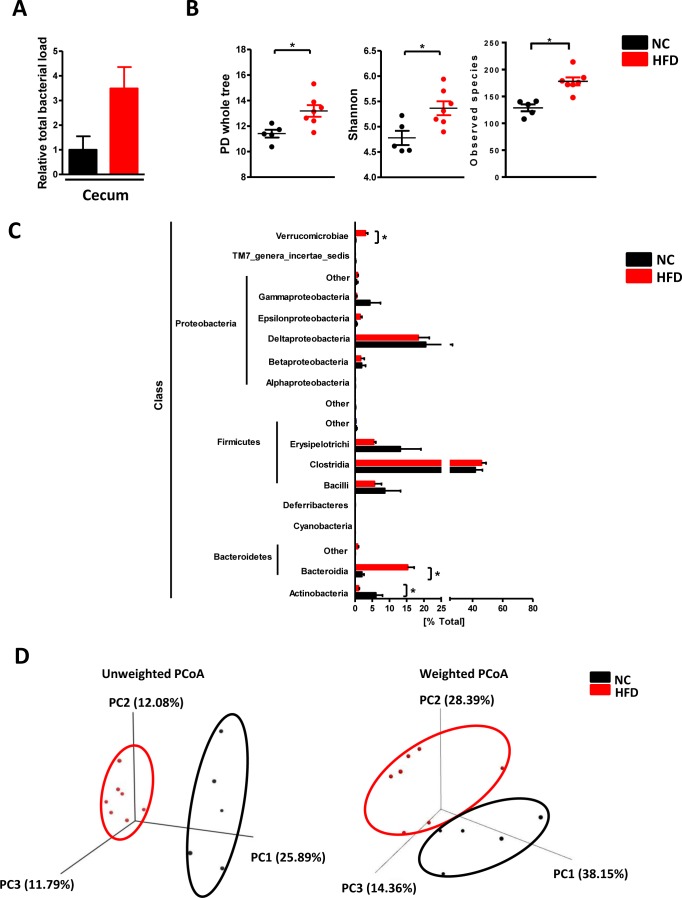
HFD caused enteric dysbiosis C57BL/6 male mice were fed with a normal chow diet (*n* = 5) or high fat diet (*n* = 7) for 17 weeks. **A**. total bacterial load in the cecum; **B**. the value of PD whole tree, Shannon and Observed species diversity; **C**. the percentage of indicated class; **D**. the PCoA analysis. Results are expressed as mean ± SEM. *p < 0.05.

Next, we determined how HFD affects gut microbiome. Total bacterial load in the cecum displayed an increasing trend after HFD but without significance (Figure 2A). To further explore the bacterial composition in the gut, we performed 16S sequencing for the DNA from cecal content. As shown in Figure 2B, the values of alpha diversity, which is represented by PD whole tree, Shannon and Observed species, were all increased after HFD compared with NC. Moreover, the percentage of Actinobacteria, Bacteroidia, and Verrucomicrobiae were also significantly altered in HFD mice (Figure 2C). Both unweighted and weighted principal components analysis (PCoA) revealed the NC and HFD clusters could be completely separated (Figure 2D). These data confirmed that HFD could disrupt enteric eubiosis.

**Figure 3:**
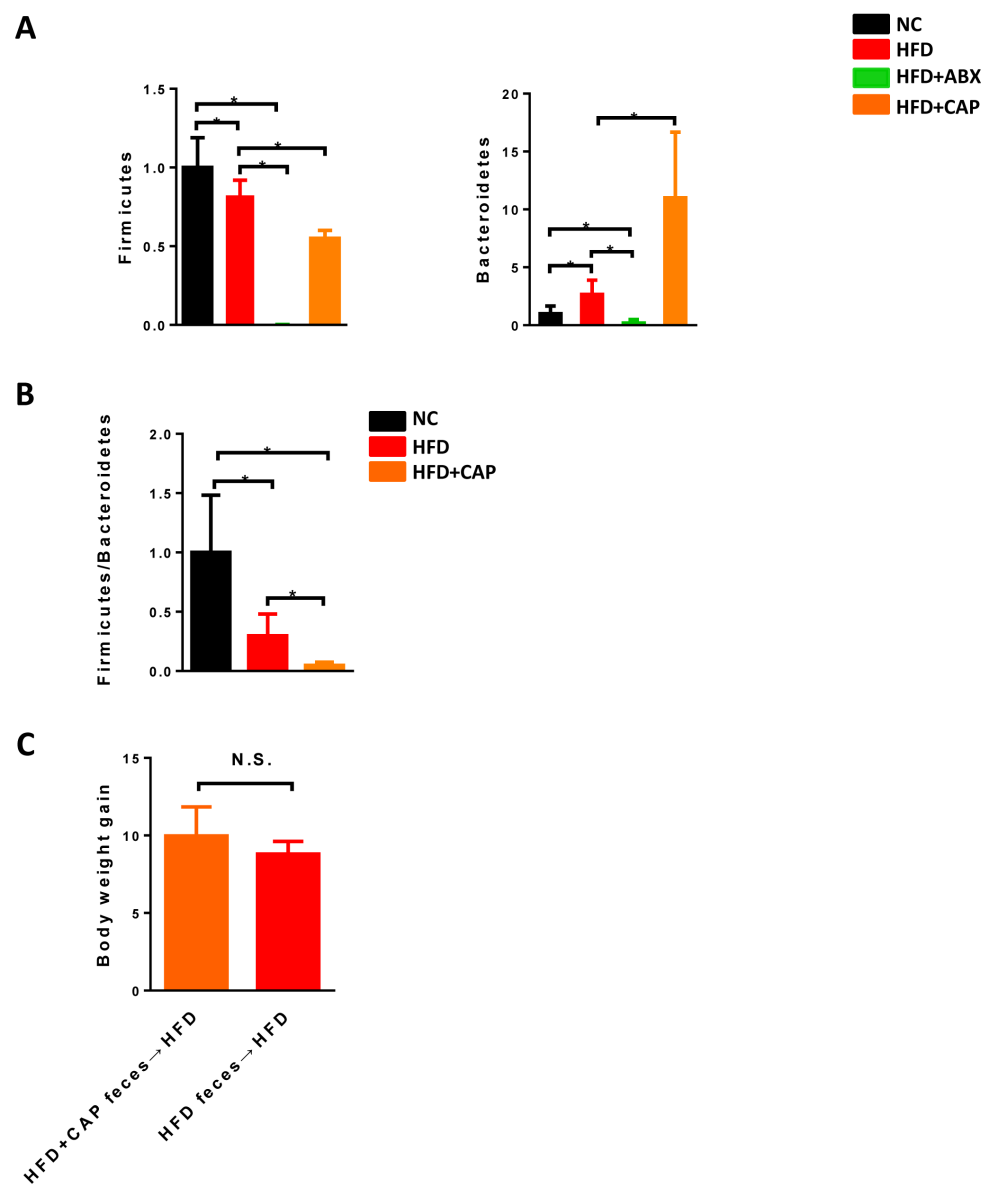
Effects of capsaicin on gut microbiota C57BL/6 male mice were fed with a normal chow diet (*n* = 5) or high fat diet (*n* = 5-9). **A**. relative abundance of Firmicutes, Bacteroidetes and **B**. Firmicutes/Bacteroidetes ratio in the cecum; **C**. Body weight gain after 12 weeks HFD feeding. Results are expressed as mean ± SEM. *p < 0.05.

In addition, capsaicin had been reported to alter gut microbial composition [19]. Figure 3A-3B confirmed that capsaicin could affect the gut microbiota as evidenced by decreased Firmicutes, increased Bacteroidetes and impaired Firmicutes/Bacteroidetes ratio in the cecum of HFD and capsaicin co-treated mice compared with HFD mice. To test whether altered microbiota induced by capsaicin exhibited physiological effects, we transplanted HFD + Cap-fed mice feces into HFD-fed mice, and monitored the body weight change. Figure 3C showed that the feces from HFD + CAP-fed mice could not reduce body weight gain induced by high fat diet. This result suggested that although the capsaicin can cause the gut microbial composition change, such alteration may not display physiological functions.

### Antibiotics, but not capsaicin, dramatically reduced intestinal inflammation and leakiness induced by HFD

**Figure 4:**
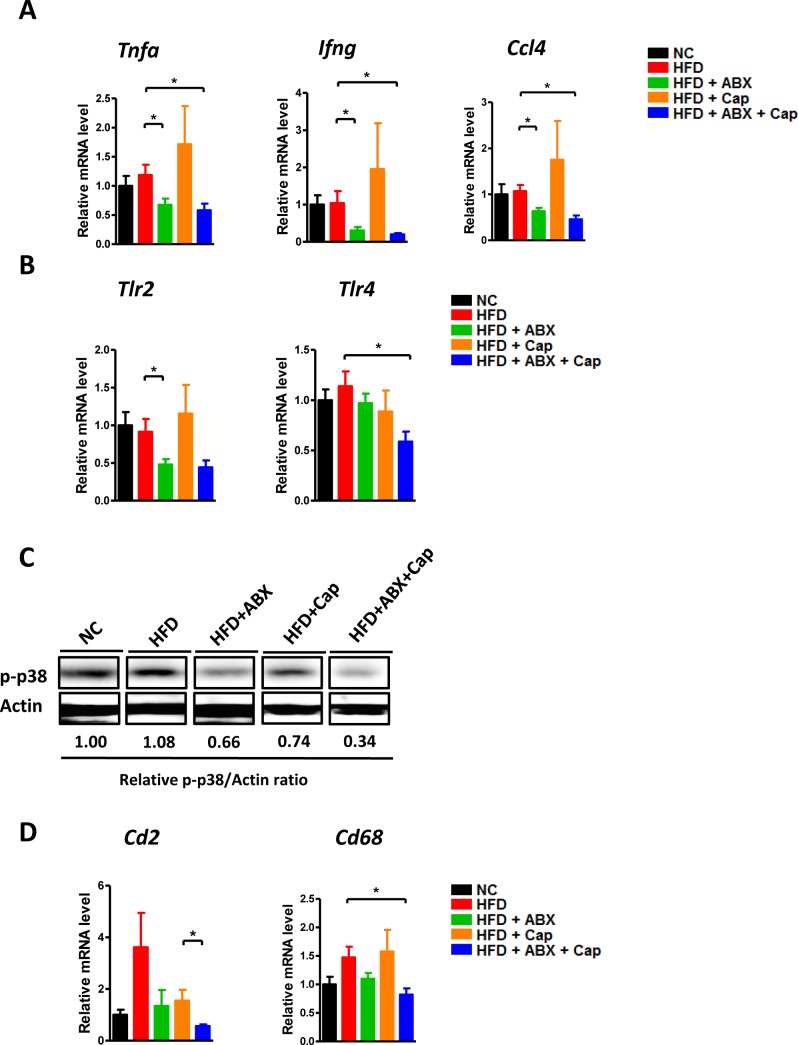
Antibiotics reduced intestinal inflammation C57BL/6 male mice were divided by 5 groups: NC (*n* = 6); HFD (*n* = 10); HFD + ABX (*n* = 10); HFD + Cap (*n* = 6); HFD + ABX + Cap (*n* = 6). **A**. *Tnfα, Ifng* and *Ccl4* mRNA level in the ileum; **B**. *Tlr2, Tlr4* mRNA level in the ileum; **C**. p-p38 protein level in the ileum; **D**. *Cd2* and *Cd68* mRNA level in the ileum. Results are expressed as mean ± SEM. *p < 0.05.

Because intestinal pathophysiological status plays a key role in HFD induced abnormalities, we examined how capsaicin and antibiotics affect gut (ileum) during HFD. First, both Firmicutes and Bacteroidetes which were the 2 main phyla in the gut were almost undetected in antibiotics treatment group, indicating the antibiotics treatment was successful (Figure 3A). Intestinal inflammation characterized by the expression of key cytokines/chemokines, such as *Tnfa, Ifng* and *Ccl4* was dramatically decreased in both HFD + ABX and HFD + Cap + ABX groups, whereas capsaicin did not affect the expression of these inflammatory factors (Figure 4A). We further measured the levels of *Tlrs* (*Tlr2, Tlr4*), MAPK (p-38) and *CDs* (*Cd2* and *Cd68*), key molecules involved in inflammatory response. *Tlr4* was significantly decreased in HFD fed animals treated with Cap + ABX but not in animals treated with antibiotics or capsaicin alone. *Tlr2* and *Cd68* exhibited a decreasing trend in both HFD + ABX and HFD + Cap + ABX groups but not in HFD + Cap mice. Antibiotics or capsaicin alone reduced the expression of *Cd2* and p-p38 while co-treatment further decreased the expression of these molecules (Figure 4B-4D). These data indicate that antibiotics significantly reduced gut inflammation while capsaicin's benefits were limited.

**Figure 5:**
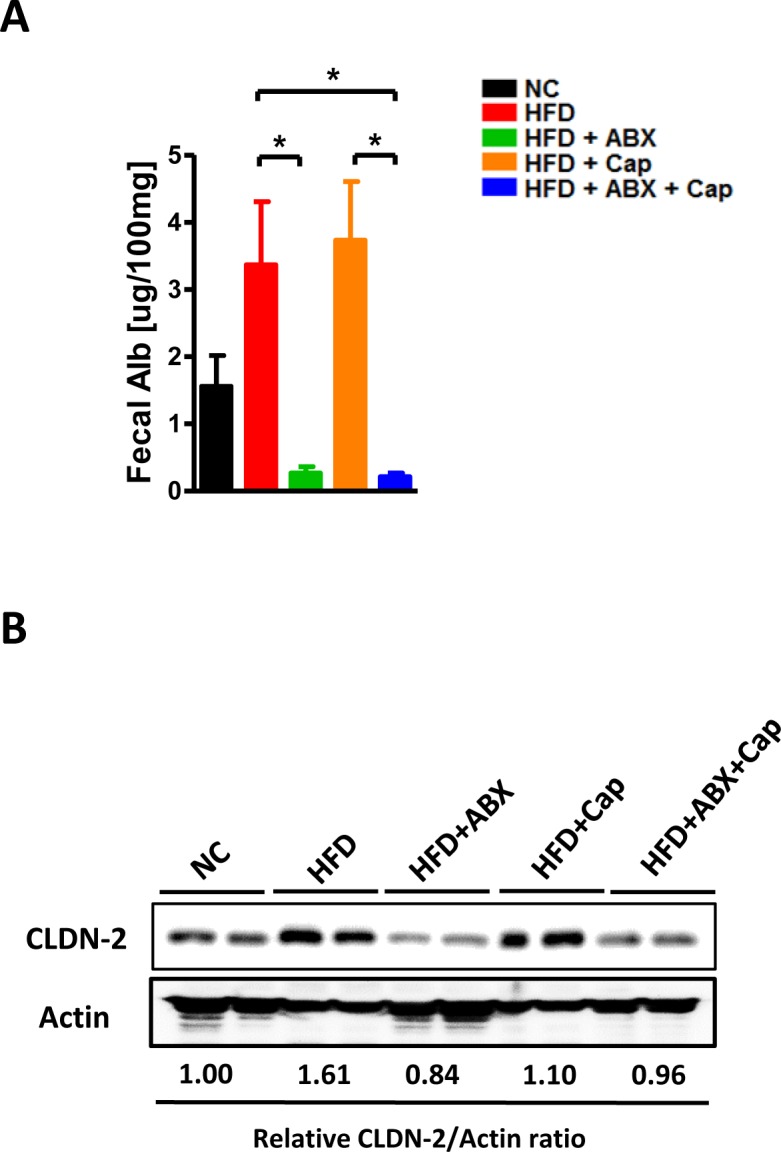
Antibiotics improved gut barrier function C57BL/6 male mice were divided by 5 groups: NC (*n* = 6); HFD (*n* = 10); HFD + ABX (*n* = 10); HFD + Cap (*n* = 6); HFD + ABX + Cap (*n* = 6). **A**. Fecal albumin content; **B**. Claudin2 protein level in the ileum. Results are expressed as mean ± SEM. *p < 0.05.

Intestinal inflammation is closely linked with gut barrier function, which is thought to influence extra-intestinal organ diseases [20–21]. Gut leakiness, indicated by fecal albumin level [22–23], showed that both ABX and Cap + ABX co-treatment reduced gut barrier disruption, but capsaicin alone provided limited effects (Figure 5A). The expression of the pore-forming tight junction protein Claudin2 (CLDN2) confirmed this finding (Figure 5B). Taken together, these results clearly suggest that antibiotics dramatically reduced HFD-linked gut inflammation and leakiness while capsaicin showed only mild protective effects, Cap + ABX co-treatment greatly improved gut abnormalities.

### Capsaicin, but not antibiotics, increased PPARα expression in adipose tissue

**Figure 6:**
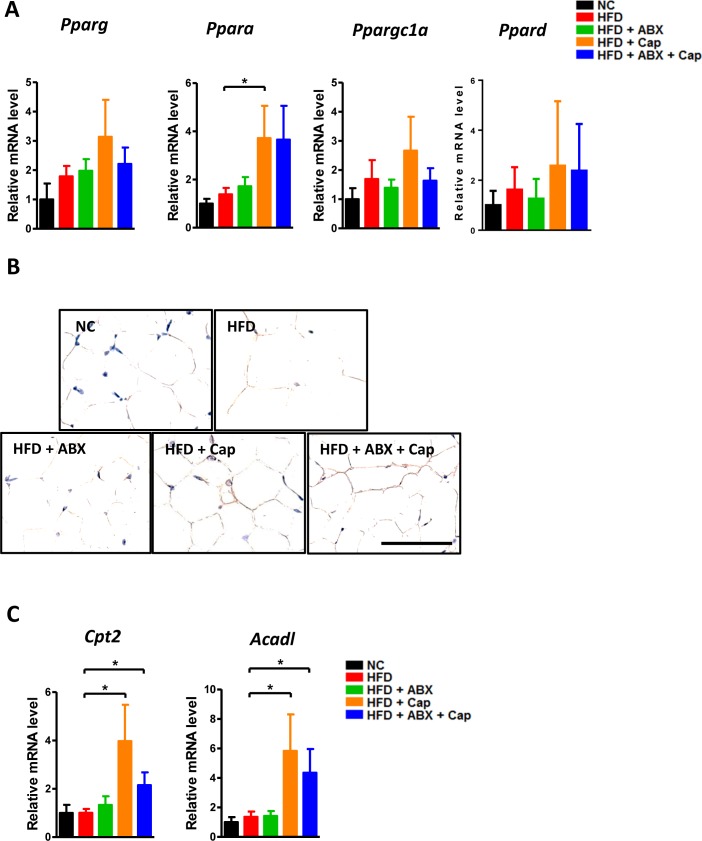
Capsaicin enhanced PPARα expression in the adipose tissue C57BL/6 male mice were divided by 5 groups: NC (*n* = 6); HFD (*n* = 10); HFD + ABX (*n* = 10); HFD + Cap (*n* = 6); HFD + ABX + Cap (*n* = 6). **A**. *Pparg, Pparα, Ppargc1a* and *Ppard* mRNA level in the white adipose tissue; **B**. PPARα immunohistochemistry staining; **C**. *Cpt2* and *Acadl* mRNA level. Scale bar, 50 μm. Results are expressed as mean ± SEM. *p < 0.05.

In addition to the gut, adipose tissue plays an important role in HFD-induced pathological changes. In particular, expression of the PPARs could represent the modulatory function of the white fat tissue [24–25]. We examined mRNA level of the PPARs in the perigonadal visceral adipose tissue in each group. As shown in Figure 6A, *Ppara, Pparg, Ppargc1a*, and *Ppard* all showed an increasing trend in the HFD + Cap group compared with HFD mice. However, only the increase in PPARα mRNA level reached statistical significance. Immunohistochemistry data confirmed the gene expression result (Figure 6B). *Cpt2* and *Acadl*, two PPARα target genes, displayed significantly higher expression in both HFD + Cap and HFD + Cap + ABX groups than in untreated HFD mice, whereas ABX single administration did not markedly alter the expression of either gene (Figure 6C). Our results suggest that capsaicin, but not antibiotics, dramatically induced PPARα and related target gene expression.

### Capsaicin and antibiotics synergistically reduced HFD induced obesity

**Figure 7:**
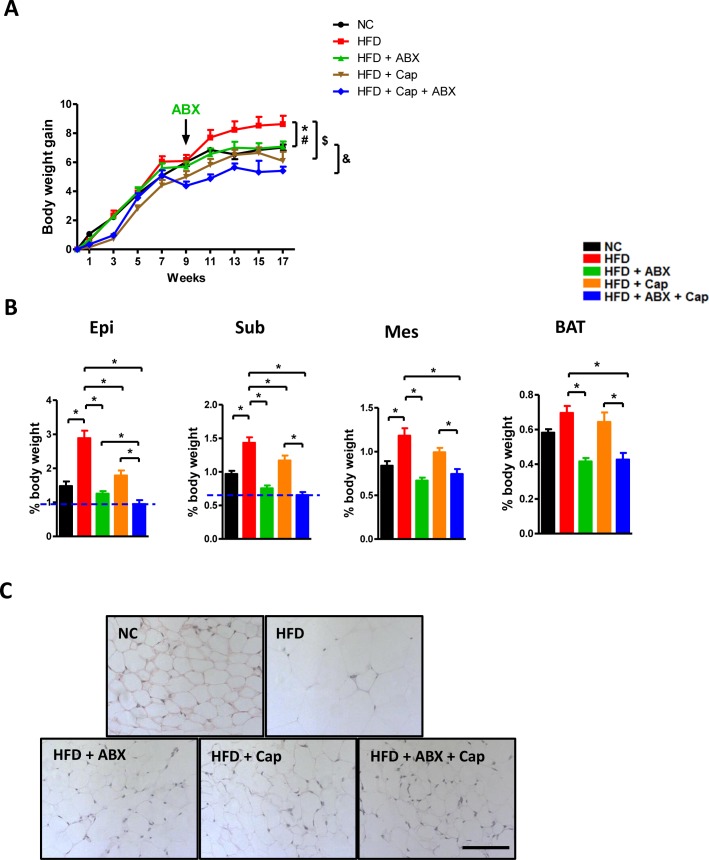
Capsaicin and antibiotics synergistically reduced HFD induced obesity C57BL/6 male mice were divided by 5 groups: NC (*n* = 6); HFD (*n* = 10); HFD + ABX (*n* = 10); HFD + Cap (*n* = 6); HFD + ABX + Cap (*n* = 6). **A**. Body weight gain; **B**. The index of epididymal (Epi), subcutaneous (Sub), mesenteric (Mes) white adipose and brown adipose tissue (BAT); **C**. HE staining of epididymal white adipose tissue. Scale bar, 100 μm. Results are expressed as mean ± SEM. *p < 0.05 HFD *vs* NC; ^#^*p* < 0.05 HFD *vs* HFD + ABX; ^$^*p* < 0.05 HFD *vs* HFD + Cap;^&^*p* < 0.05 HFD + ABX + Cap *vs* HFD + ABX in panel A; *p < 0.05 in panel B.

After 17 weeks, HFD fed mice exhibited a significantly higher body weight compared with all other groups. More importantly, the body weight gain in HFD + ABX mice was similar to NC mice while that in HFD + Cap and HFD + Cap + ABX animals was even less than in NC mice (Figure 7A). It was noted that before antibiotics treatment, there was no difference in body weight gain between HFD and HFD + ABX mice. The difference appeared in response to antibiotics administration. Additionally, HFD + Cap + ABX mice showed the lowest body weight gain among all groups. The index of epididymal, subcutaneous, mesenteric, and brown adipose tissue was markedly reduced in HFD + ABX, HFD + Cap, and HFD + Cap + ABX compared with untreated HFD mice (Figure 7B). Notably, HFD + Cap + ABX showed the lowest index of epididymal and subcutaneous fat; the epididymal fat index was significantly lower in HFD + Cap + ABX than HFD + ABX mice. Hematoxylin and eosin (HE) staining for epididymal adipose tissue confirmed the above findings (Figure 7C). Our data clearly demonstrate capsaicin and antibiotics synergistically reduced HFD induced obesity.

### Capsaicin and antibiotics synergistically reduced HFD induced fatty liver development

**Figure 8:**
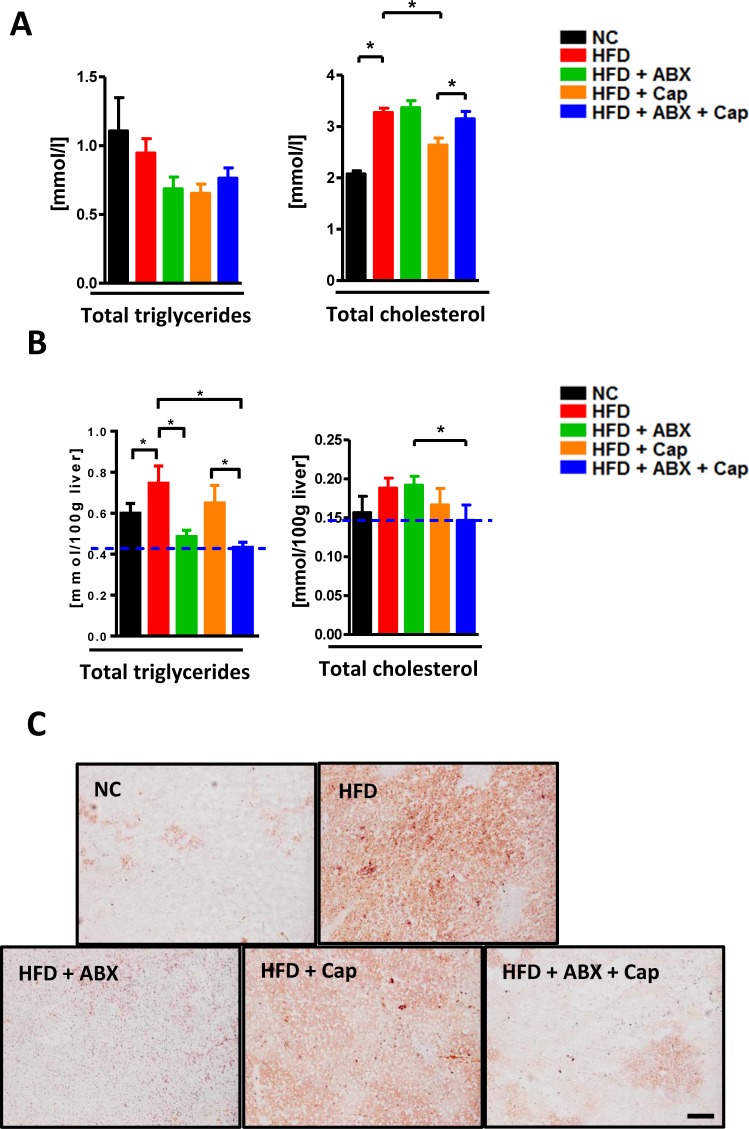
Capsaicin and antibiotics synergistically reduced HFD induced fatty liver C57BL/6 male mice were divided by 5 groups: NC (*n* = 6); HFD (*n* = 10); HFD + ABX (*n* = 10); HFD + Cap (*n* = 6); HFD + ABX + Cap (*n* = 6). **A**. Plasma triglyceride and cholesterol level; **B**. Hepatic triglyceride and cholesterol level; **C**. Oil red O staining of liver. Scale bar, 100 μm. Results are expressed as mean ± SEM. *p < 0.05.

Hepatic steatosis is recognized as a principle pathological change during HFD induced metabolic disorder. We investigated how capsaicin and antibiotics affect fatty liver development in HFD-fed mice. Plasma triglyceride and cholesterol levels were reduced in HFD + Cap mice compared to HFD mice, while antibiotics did not markedly affect the plasma cholesterol level (Figure 8A). However, antibiotics, as well as capsaicin, markedly reduced triglyceride accumulation in the liver. Cholesterol level was not dramatically altered by antibiotics or capsaicin alone. Interestingly, livers from HFD + Cap + ABX showed the lowest triglyceride and cholesterol levels of all groups (Figure 8B). Oil red O staining in the liver confirmed the hepatic triglyceride data (Figure 8C). These results indicate that capsaicin and antibiotics co-treatment may maximally ameliorate HFD-caused fatty liver.

### Capsaicin and antibiotics synergistically reduced HFD induced insulin resistance

**Figure 9:**
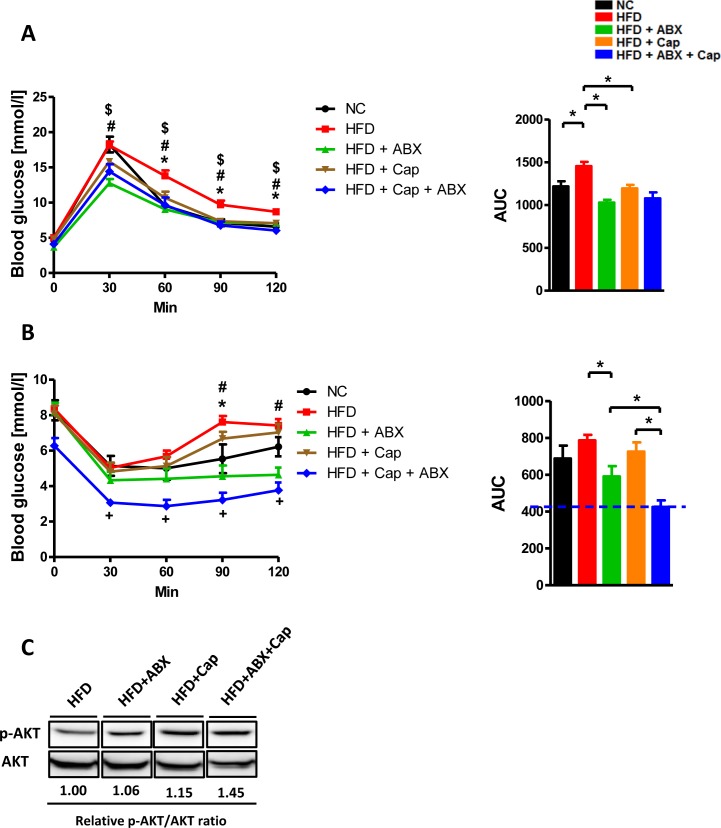
Capsaicin and antibiotics synergistically reduced HFD induced insulin resistance C57BL/6 male mice were divided by 5 groups: NC (*n* = 6); HFD (*n* = 10); HFD + ABX (*n* = 10); HFD + Cap (*n* = 6); HFD + ABX + Cap (*n* = 6). **A**. GTT (intraperitoneal) and the area under curve (AUC) of each group; **B**. ITT (intraperitoneal) and the area under curve (AUC) of each group. **C**. p-Akt, Akt protein levels in the perigonadal visceral adipose tissue. Results are expressed as mean ± SEM. *p < 0.05 HFD *vs* NC; ^#^*p* < 0.05 HFD *vs* HFD + ABX;^$^*p* < 0.05 HFD *vs* HFD + Cap in panel A (left); *p < 0.05 HFD *vs* NC; ^#^*p* < 0.05 HFD *vs* HFD + ABX;^+^*p* < 0.05 HFD + ABX + Cap *vs* HFD + Cap in panel B (left);*p < 0.05 in panel A,B (right).

We finally examined how capsaicin and antibiotics affect glucose homeostasis following HFD. The intraperitoneal glucose tolerance test (GTT) experiment demonstrated that HFD induced glucose tolerance was markedly improved by capsaicin, antibiotics, or co-treatment (Figure 9A). Consistent with this, untreated HFD mice exhibited the worst responsiveness to insulin of all groups (Figure 9B). Notably, mice treated with both antibiotics and capsaicin exhibited the greatest responsiveness to insulin among all groups. Insulin resistance is characterized by the failure of insulin to repress the expression of gluconeogenic genes, which was mainly mediated by the Akt signaling pathway [26]. p-Akt/Akt ratio in the perigonadal visceral adipose tissue was higher in HFD + Cap + ABX mice compared to HFD mice (Figure 9C). These data indicate that co-administration of antibiotics and capsaicin results in the best improvement of HFD-induced insulin resistance.

## DISCUSSION

It is well established that development of most diseases depends on a variety pathways including multiple organ interactions [27–29]. Hence, cumulative effects provided by different therapeutic approaches targeting distinct disease mechanisms should be better than any single treatment. Indeed, co-treatment has been applied to many disease models. For example, in ApoE deficient mice, combined treatment with ERK1/2 inhibitor and LXR ligand synergistically reduced atherosclerosis, which may function as a novel therapy [30]. HFD induced obesity and metabolic syndrome are recognized as the classic disease with multiple organ interactions. However, few pieces of evidence revealed the potential cumulative benefit of targeting different organs. In the present study, we first supported this possibility by co-administering capsaicin and antibiotics. Capsaicin has many beneficial effects including anti-obesity action [14, 31–33], however, its detailed mechanism is not clear. Here, we showed that capsaicin significantly elevated PPARα expression in adipose tissue after HFD. PPARα is a key gene that modulates lipid deposition, and several direct pieces of evidence indicate that selective PPARα activation significantly reduces body weight gain and insulin resistance in rodents after HFD by regulating fat-metabolism associated gene expression [34]. Cpt2 and Acadl, both regulated by PPARα [35–36], are important metabolic molecules involved in fatty acid oxidation [37–38]. It was reported that the elevated expression of these genes was associated with increased body weight gain, glucose tolerance, and fatty liver induced by HFD [39–40]. In the present study, only HFD + Cap and HFD + Cap + ABX groups displayed increased expression of Cpt2 and Acadl, which demonstrated that capsaicin, but not antibiotics, targeted PPARα expression in adipose tissue.

Diet is a major factor driving the composition and metabolic activity of the gut microbiota, and it may also affect intestinal barrier function and result in metabolic endotoxemia [41]. Gut microbiota is recognized as a major contributor to HFD induced obesity and metabolic disorder [8–10, 42]. Antibiotics markedly reduce obesity development in mice and improved gut pathophysiological status is believed to be the main underlying mechanism. We further confirmed this point. First, antibiotics reduced intestinal inflammation, which is the main characterization of intestinal abnormalities. Improvement was not confined to reduced cytokine expression, but also included reduced expression of inflammation-related signaling pathway components like p-p38 protein. Impaired inflammatory response was closely linked with gut leakiness [43]. Inflammatory factors could also directly disrupt tight junctions protein and promote barrier dysfunction [44]. Furthermore, our results suggest that antibiotics significantly attenuated HFD induced gut leakiness by preventing the elevation of the pore-forming tight junction protein Claudin2 (CLDN2). Disrupted gut barrier integrity is believed to promote NAFLD associated pathologies such as fatty liver and insulin resistance progression [3]. Thus, our results suggested that by inducing inflammatory response related signaling pathway such as p-p38, gut microbiota would induce the intestinal inflammation first and in turn enhanced the gut permeability, finally promoted NAFLD development during HFD feeding. These results suggest that the beneficial actions of antibiotics mainly target the intestine. Notably, animals co-treated with capsaicin and antibiotics exhibited all of the beneficial effects in both intestine and adipose tissue. Finally, co-treatment also resulted in the lowest body weight gain, epididymal and subcutaneous adipose tissue index, and hepatic total triglyceride level, and the best responsiveness to insulin. We noted that there was no significant difference between the effects of antibiotics alone and co-treatment on many phenotypic parameters. This may be because antibiotics already dramatically reduced these parameters to even lower than normal chow animals, limiting the reduction space for co-treatment.

The translational significance of the current study is to provide a novel combinational approach for the treatment of HFD-associated abnormalities. First, dietary capsaicin benefits adipose tissue by promoting fatty acid oxidation associated gene expression and reduces fat accumulation in response to HFD. Second, a strategy that maintains intestinal homeostasis such as antibiotics, prebiotics, and probiotics may further attenuate insulin resistance and fatty liver development by reducing gut barrier disruption. This combined approach may provide the maximum beneficial effect if used in patients that suffer from obesity associated non-alcoholic fatty liver disease.

## MATERIALS AND METHODS

### Animals

Male 6-8 week-old specific pathogen free (SPF) C57BL/6 mice were used in this work. Mice were divided into 5 groups: (1) normal chow (NC): fed with a low fat (10% calories from fat) diet for 17 weeks; (2) high fat diet (HFD): fed with a high fat diet (60% calories from fat) for 17 weeks; (3) high fat diet + antibiotics (HFD + ABX): fed with a high fat diet and gavage with antibiotics (vancomycin, 100mg/kg; neomycin, 200mg/kg; metronidazole, 200mg/kg; ampicillin, 200mg/kg, start from 9th week, once a day in 9th week, then three times a week until 17th week); (4) high fat diet + capsaicin (HFD + Cap): fed with a high diet contained 0.015% capsaicin in the first 10 weeks, capsaicin concentration increased into 0.02% from 11th week to 17th week; (5) high fat diet + antibiotics + capsaicin (HFD + ABX + Cap): fed with a high fat diet and treated with antibiotics and capsaicin as described above. All mice that received the capsaicin were normal and did not show any physiological abnormality. For the fecal transplantation experiment, male 6-8 week-old C57BL/6 mice were fed high fat diet for 12 weeks. Stool was firstly collected from HFD-fed mice and HFD + Cap fed mice, respectively, then transplanted into the corresponding receiver's cages three times a week started from 1^st^ week. GTT and ITT were performed 2-3 weeks before sacrifice. For GTT experiment, mice were fasted for 16h and 1g/kg glucose was administered i.p. Blood glucose concentration was measured at 0min, 30min, 60min, 90min, and 120min. For ITT experiment, mice were fasted for 6h and 0.35U/kg insulin was administered i.p. Blood glucose concentration was measured at 0min, 30min, 60min, 90min, and 120min. All mice had free access to food and water and were maintained in a temperature-controlled colony room on a 12:12-h light/dark cycle. All experimental procedures were in compliance with the National Institutes of Health guidelines and were approved by the local Animal Care and Use Committee of the Southern Medical University.

### Microbe analysis

Cecum content was resuspended in PBS containing 0.5% Tween 20 and further subjected to a -80°C/60°C cycle three times to destroy the membrane. DNA extraction and cecal total bacteria load were further analyzed as described [45]. Quantitative real time PCR was performed using 16s rRNA primers: 5′- GTGSTGCAYGGYTGTCGTCA-3′; 5′- ACGTCRTCCMCACCTTCCTC-3′; Firmicutes primers: 5′-GGAGYATGTGGTTTAATTCGA -3′; 5′-AGCTGACGACAACCATGCAC-3′; Bacteroidetes primers: 5′-GGCGACCGGCGCACGGG -3′; 5′-GRCCTTCCTCTCAGAACCC-3′. A microbial diversity analysis was performed [46]. Briefly, we firstly amplified the 16S rRNA gene V4 region by PCR and using the Ion Torrent sequencing platform for further sequencing. The raw sequences were quality-controlled using QIIME (1.9.1). Then the sequences were demultiplexed and clustered into species-level (97% similarity) operational taxonomic units (OTUs). Finally, strain composition analysis, alpha diversity analysis and beta diversity analysis were also performed by QIIME. LEfSe (LDA Effect Size, http://huttenhower.sph.harvard.edu/galaxy/) was used for discriminative taxa determination.

### Real time PCR

Total RNA was extracted using Trizol according to the manufacturer's instructions. A reverse transcription reaction was carried out by reverse transcriptase (TOYOBO) according to the manufacturer's instructions. The real-time PCR reaction was carried out on ABI 7500 real-time PCR system with cDNA sample containing 1×SYBR Green PCR master mix (TOYOBO) using primer sequences obtained from NIH qPrimerDepot. Relative expression in comparison with 18s rRNA was calculated by the comparative CT method.

### Histology analysis

Tissue was collected and fixed in 10% buffered formalin. The sample was then embedded in paraffin and sliced into 5-μm-thick sections. Hematoxylin and eosin (HE) staining was performed and analyzed by microscopy. Immunohistochemistry was performed using the primary antibodies for PPARα and TRPV1 (Proteintech). Frozen sections (8μm) were used for Oil Red O staining.

### Protein analysis and biochemical analysis

Tissue protein was extracted using a commercial lysis buffer (KeyGene). Western blot was performed using the primary antibodies for Claudin-2 (Invitrogen), p-p38, (Bioworld technology), p-AKT, AKT (ABcolonal), β-Actin (Proteintech). Fecal albumin was determined by ELISA (Bethyl Labs). Triglycerides and cholesterol concentrations were measured on the automatic biomedical analyzer (Roche) using commercial kit.

### Statistical analysis

Results are expressed as mean ± SEM. Two-way ANOVA analysis followed by post hoc with two-tailed *t*-tests was used to analyze the capsaicin and antibiotics data. To analyze variable difference among NC mice and HFD mice, 2-tailed unpaired Student *t* test was used. A p-value less than 0.05 was considered to be statistically significant.
